# Heterogeneous dynamics in the curing process of epoxy resins

**DOI:** 10.1038/s41598-021-89155-x

**Published:** 2021-05-17

**Authors:** Taiki Hoshino, Yasushi Okamoto, Atsushi Yamamoto, Hiroyasu Masunaga

**Affiliations:** 1 RIKEN SPring-8 Center, 1-1-1, Kouto, Sayo-cho, Sayo-gun, Hyogo, 679-5148 Japan; 2 DENSO CORPORATION, 1-1, Showa-cho, Kariya, Aichi 448-8661 Japan; 3 Japan Synchrotron Radiation Research Institute/SPring-8, 1-1-1, Kouto, Sayo-cho, Sayo-gun, Hyogo, 679-5198 Japan

**Keywords:** Structure of solids and liquids, Polymer chemistry, Glasses, Polymers

## Abstract

Epoxy resin is indispensable for modern industry because of its excellent mechanical properties, chemical resistance, and excellent moldability. To date, various methods have been used to investigate the physical properties of the cured product and the kinetics of the curing process, but its microscopic dynamics have been insufficiently studied. In this study, the microscopic dynamics in the curing process of a catalytic epoxy resin were investigated under different temperature conditions utilizing X-ray photon correlation spectroscopy. Our results revealed that the temperature conditions greatly affected the dynamical heterogeneity and cross-linking density of the cured materials. An overview of the microscopic mechanism of the curing process was clearly presented through comparison with the measurement results of other methods, such as ^1^H-pulse nuclear magnetic resonance spectroscopy. The quantification of such heterogeneous dynamics is particularly useful for optimizing the curing conditions of various materials to improve their physical properties.

## Introduction

Intermittent dynamics appears in soft solids such as polymer gels, colloidal gels, and glass formers at the microscale^1–7^. Understanding intermittent dynamics has become increasingly important for the elucidation of nonequilibrium phenomena, such as aging and stress relaxation^8,9^. Such intermittent dynamics indicate the coexistence of fast and slow dynamics regions, which can be caused by density fluctuation of the cross-linking network in the thermosetting process of epoxy resins. It can considerably influence the final mechanical properties of cured materials. Thermoset epoxy resins are indispensable tools for modern industry because of their excellent mechanical properties, high adhesion to various substrates, and excellent heat and chemical resistances. They are used in various fields as versatile adhesives, fiber reinforced materials, and high-performance coatings^10–12^. In the production process, efficient energy use in the thermosetting process is becoming increasingly important, for which optimization of curing conditions is required. Thus, it is necessary to properly understand the kinetics of the thermosetting process.

The thermal curing reaction is typically initiated by mixing two reactive components together and elevating the temperature to cause an oligomerization and cross-linking reaction. Since the cross-linking network is strongly correlated to the main properties of the cured materials, curing kinetics have attracted considerable interest and have been vigorously investigated using various methods. The reaction mechanism has been studied through the analysis of reaction kinetics by investigating the isothermal hardening process with differential scanning calorimetry^13–16^. The reaction kinetics have also been investigated using near-infrared spectroscopy and Raman spectroscopy^14,17,18^. The kinetics of mechanical properties have been examined using rheological methods. The divergence of the shear modulus near the gel point and the behavior of the frequency-dependent shear modulus have greatly contributed to the understanding of the relationship between the reaction mechanism and the macroscopic physical properties^19–22^. The effect of temperature on the isothermal curing reaction is fairly complex^23–25^. Various mathematic kinetic models of chemical processes have been used to study the effect of curing temperature on the isothermal curing process of epoxy resins, and they reasonably explain that the reaction is faster, and the final reaction degree is higher because of the higher mobility of the molecules at high temperatures. This results in a higher glass transition temperature (*T*_g_) of the curing materials^14,26–29^. However, in the industrial field, it is known that there are different types of epoxy resin, in which the cured product first cured at a low temperature and post-cured at a high temperature has a higher *T*_g_ than does a product that has been cured at a high temperature from the beginning. The value of *T*_g_ is known to have a deep correlation with the cross-linking density, which also directly correlates with the heterogeneity of the cross-linking structure^30–33^. The heterogeneity of cross-linking networks has been sensitively detected in dynamic fluctuations as reported in polymer network gels using dynamic light scattering measurements^34^.

Heterogeneous dynamic fluctuations, including intermittent ones, have a deep correlation with the cross-linking structure. Thus, investigating the microscopic dynamics of the cross-linking process will provide important insights into the curing mechanism. X-ray photon correlation spectroscopy (XPCS) is a measurement technique that permits the observation of the dynamics of a sample at the microscale based on the temporal fluctuation of the scattering intensity of partially coherent X-rays^35,36^. XPCS is a promising technique for dynamically studying the kinetics of cross-linking systems at the microscale. Czakkel and Madsen investigated the evolution of dynamics and structure during the formation of a cross-linked polymer gel using XPCS and small angle X-ray scattering^37^. They succeeded in associating dynamic slowing and crossover with the evolution of the structure. Begam et al. studied the kinetics of network formation and dynamics of an egg white gel using XPCS and ultrasmall-angle X-ray scattering, revealing the growth of network mesh size and heterogeneous dynamics^38^. Recently, Koga et al. performed XPCS measurements of the thermosetting process of epoxy resins containing some fillers, showing that XPCS is a powerful tool for observing the epoxy dynamics from liquid to solid states^39^. They successfully observed the evolution of the filler’s velocity during the curing process; however, owing to the effect of the densely dispersed fillers, only the ballistic motion was observed throughout the curing process, and they could not discuss the change in the movement, such as the deviation from normal Brownian motion and the fluctuation of dynamics, in detail.

**Figure 1:**
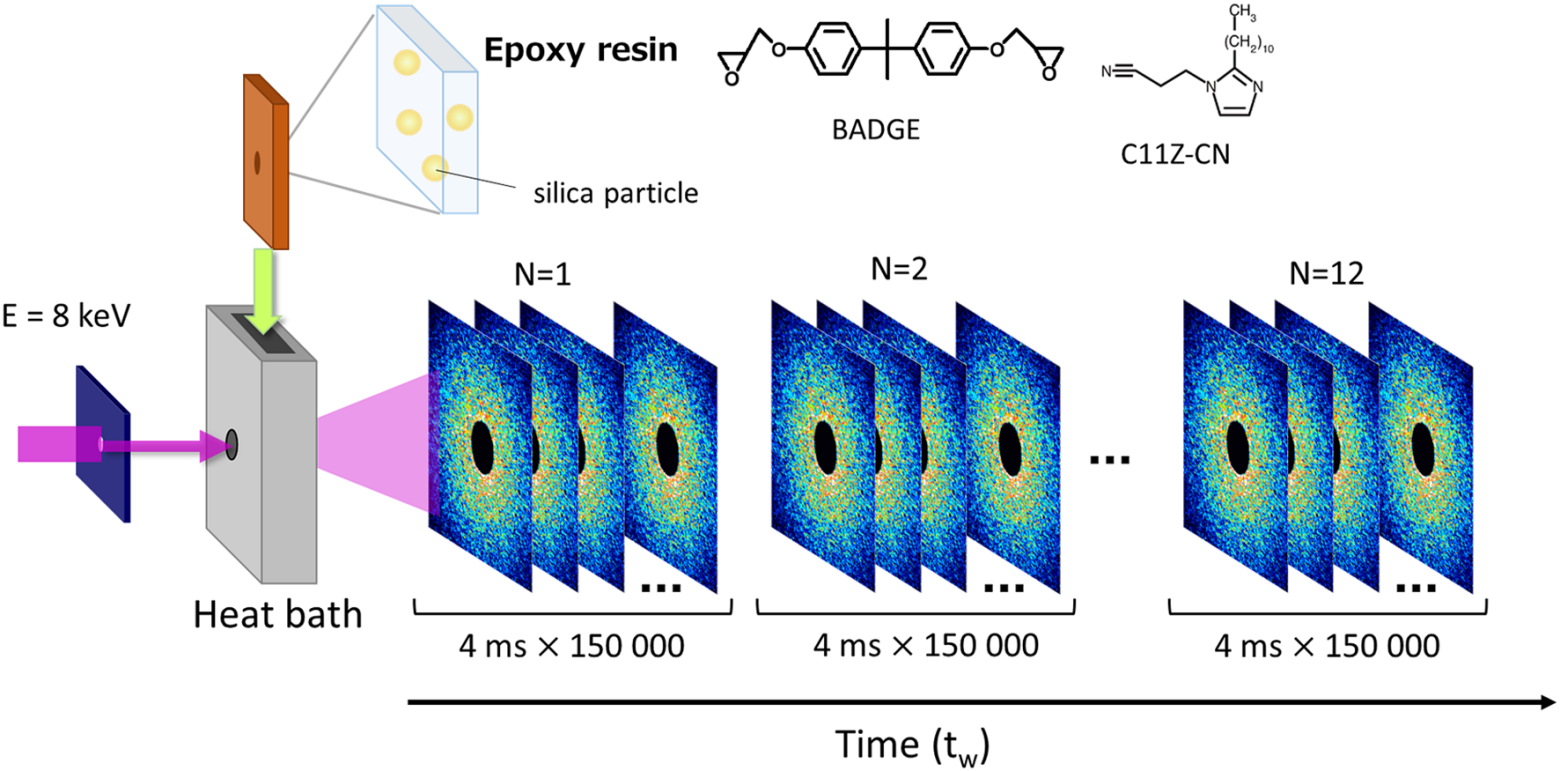
Schematic illustration of the X-ray photon correlation spectroscopy (XPCS) measurement in the curing process. A mixed solution of the main agent (bisphenol A diglycidyl ether, BADGE) and catalyst (1-(2-cyanoethyl)-2-undecylimidazole, C11Z-CN), in which nanoparticles were evenly distributed, was placed in a heat bath, and the change in dynamics was measured using XPCS in relation to the elapsed time $t_{{\text{w}}}$. The time-resolved measurement of 150,000 frames with an exposure time of 4 ms as well as the movement of the irradiation position were repeated 12 times to investigate the dynamics of the curing process for approximately 2 h. The illustration was made using Microsoft PowerPoint 2013.

In this study, to focus on the dynamics of epoxy matrix under the condition that the influence of the fillers is almost negligible, we used a simple system in which spherical particles were dispersed in the resin at a dilute concentration as a probe (The effect of silica nanoparticles were discussed in Supplementary Information with Supplementary Figs. 9 and 10). This made it possible to have a detailed discussion on the deviation from the normal Brownian motion during the curing process and the fluctuations of dynamics, including intermittent ones. We researched the microscopic dynamics of a catalytic epoxy resin during the isothermal curing process under two different temperatures: 100 and 150 °C. The dynamics of the curing process from liquid to solid state were investigated in detail, as schematically shown in Fig. 1. The prior rheological measurements indicated that both the curing process at 100 and 150 °C reached the gel point within 1 h, and solidification was almost complete (see Supplementary Fig. 1). Thus, we focused on the first 2 h of the curing process and investigated its microscopic dynamics with XPCS. Although it is difficult to observe the change of mechanical properties after solidification by rheological measurements, the microscopic dynamic fluctuation after the gelation point can be detected with XPCS. In particular, examining the temporal fluctuation of dynamic behavior enables us to discuss dynamical heterogeneity, which can be quantitatively evaluated by analyzing the fluctuation of the speckle images obtained from XPCS measurements, is one of the most important parameters for characterizing gels and glassy materials, and it can be the key to understanding the physical properties of epoxy resins^2,40–42^. Furthermore, to discuss the relationship between the dynamic behavior and the cross-linking properties, ^1^H-pulse nuclear magnetic resonance (NMR) spectroscopy measurements were performed. ^1^H-pulse NMR is one of the most promising techniques for elucidating the cross-link inhomogeneity of polymer networks^43–47^. In ^1^H-pulse NMR, the mobility of molecules is evaluated from the spin–spin relaxation time of protons. Thus, the spin–spin relaxation of tightly cross-linked segments with lower molecular mobility decays faster than that of loosely cross-linked segments.

## Results

### Kinetics of microscopic dynamics in curing process

Before providing the details of the dynamic data in the curing process studied with XPCS, we briefly introduce the important aspects of the experiment. As shown in Fig. 1, a mixed solution of the main agent and catalyst, in which probe nanoparticles (120-nm diameter) were homogeneously distributed, was placed in a heat bath, and the change in dynamics was measured using XPCS with respect to the elapsed time $t_{{\text{w}}}$. The time-resolved measurement of 150,000 frames with an exposure time of 4 ms as well as the movement of the irradiation position were repeated 12 times to investigate the dynamics of the curing process for approximately 2 h. In the XPCS measurements, the fluctuation of the scattering intensity $I\left( {{\mathbf{q}},t} \right)$ at a scattering vector ${\mathbf{q}}$ was obtained over a time series *t*, and the intensity time autocorrelation function was calculated as

1 $$ g_{2} \left( {q,t} \right) = I\left( {q,t^{\prime}} \right)I\left( {q,t^{\prime} + t} \right)/I\left( {q,t^{\prime}} \right)^{2} , $$

where $q = \left| {\mathbf{q}} \right|$ and the angle brackets indicate time averaging.

**Figure 2:**
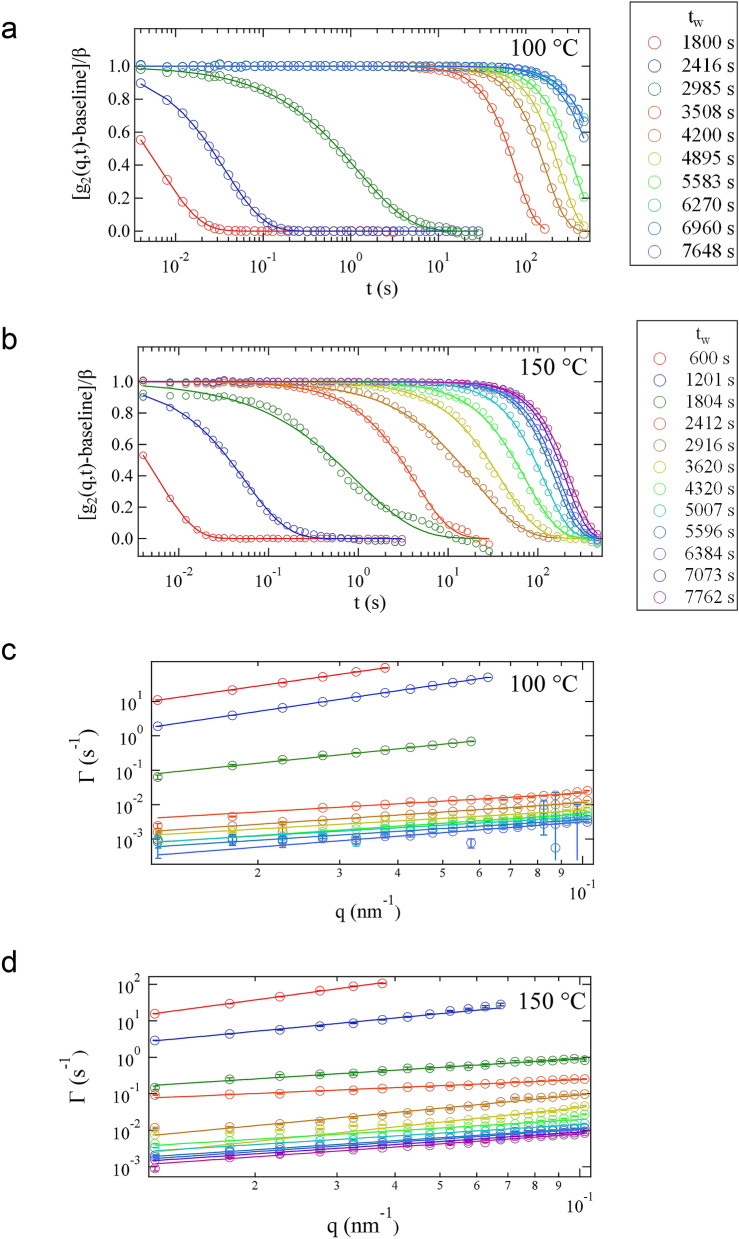
Normalized autocorrelation functions for various elapsed times at *q* = 0.0325 nm^−1^ at 100 °C (**a**) and 150 °C (**b**). The solid lines in (**a**) and (**b**) are the fitting curves from Eq. (2). The *q* dependence of ${\Gamma }$ at 100 and 150 °C is shown in (**c**) and (**d**), respectively. The solid lines in (**c**) and (**d**) are the fitting curves from the power law ${\Gamma } = Aq^{n}$.

Figure 2a,b depict the normalized correlation functions $\left[ {g_{2} \left( {q,t} \right) - {\text{baseline}}} \right]/\beta$, where $\beta$ is the speckle contrast at various elapsed times *t*_w_ at *q* = 0.0325 nm^−1^, as representative correlation functions obtained by the XPCS measurements at 100 and 150 °C, respectively. The number of frames used for the calculation of $g_{2}$ was adjusted according to the relaxation time at each *t*_w_. For example, in the initial state, $g_{2}$ was calculated every 1000 frames owing to its short relaxation time; in the latter state, $g_{2}$ was calculated at every 150,000 frames because its long relaxation time. $g_{2} \left( {q,t} \right)$ was analyzed by fitting with the following equation:

2 $$g_{2} \left( {q,t} \right) = \beta \exp \left[ { - 2({\Gamma }t} \right)^{\alpha } ] + {\text{baseline,}}$$

where ${\Gamma }$ and ${\upalpha }$ are the relaxation rate and stretched or compressed exponent, respectively. The *q* dependences of ${\Gamma }$ at 100 and 150 °C are shown in Fig. 2c,d, respectively. All the obtained *q* dependences of ${\Gamma }$ are expressed by the power law of ${\Gamma } = Aq^{n}$, where $A$ and $n$ are a constant and an exponent, respectively.

**Figure 3:**
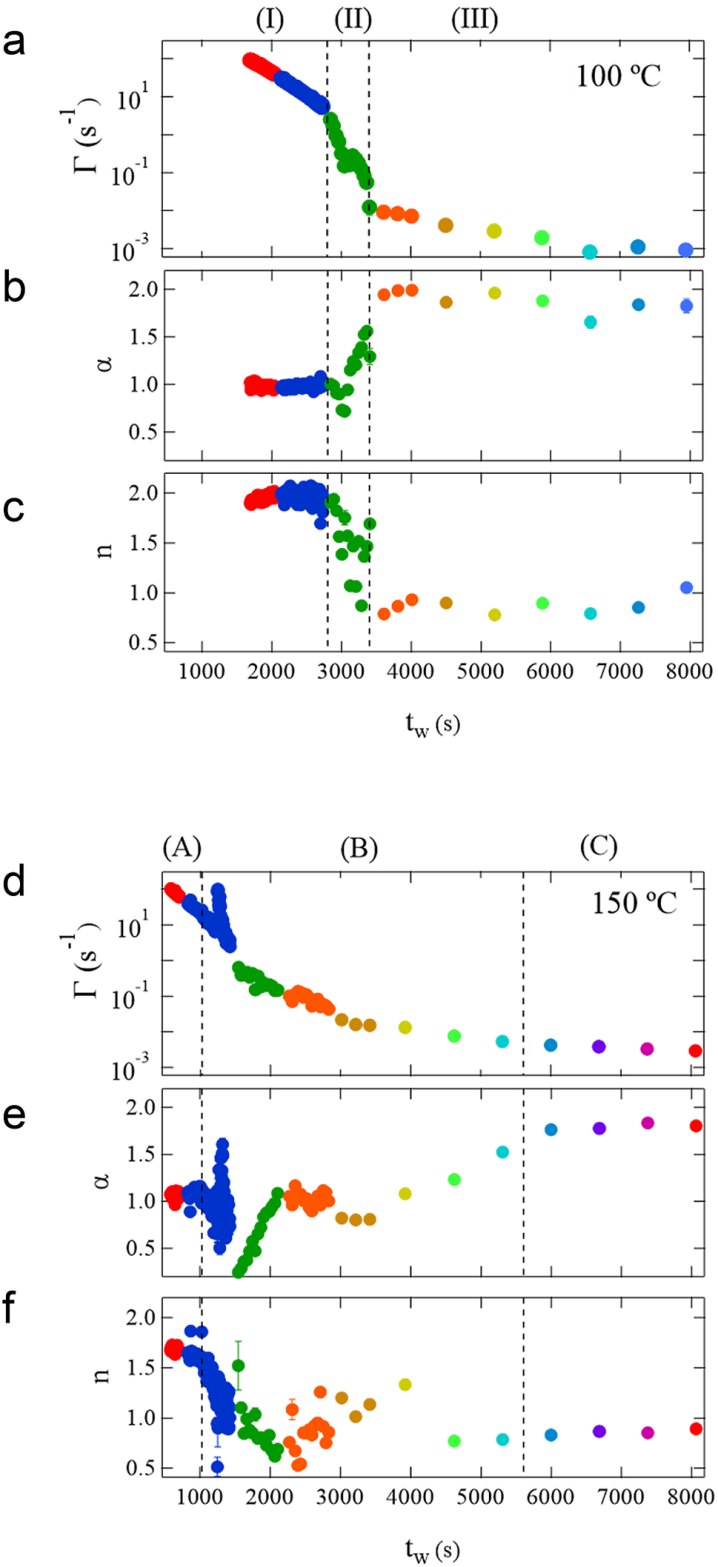
Time dependence of ${\Gamma }$ (**a**) and ${\upalpha }$ (**b**) at *q* = 0.0325 nm^−1^ obtained from the fitting analysis with Eq. (2), and $n$ (**c**) in the 100 °C curing process. Time dependence of ${\Gamma }$ (**d**) and ${\upalpha }$ (**e**) at *q* = 0.0325 nm^−1^, and $n$ (**f**) in the 150 °C curing process. The time region of each process is divided into three parts based on the behavior of the parameters: (I) $t_{{\text{w}}} < 2800{\text{ s}}$, (II) $2800{\text{ s}} < t_{{\text{w}}} < 3400{\text{ s}}$, and (III) $t_{{\text{w}}} > 3400{\text{ s}}$ in the 100 °C curing process, and (A) $t_{{\text{w}}} < 1025{\text{ s}}$, (B) $1025{\text{ s}} < t_{{\text{w}}} < 5607$, and (C) $t_{{\text{w}}} > 5607{\text{ s}}$ in the 150 °C curing process.

Figure 3 illustrates the time dependence of ${\Gamma }$ and ${\upalpha }$ at *q* = 0.0325 nm^−1^ obtained from the fitting analysis with Eq. (2) as well as $n$ in the 100 and 150 °C curing processes. First, the time dependence of the parameters in the 100 °C curing process is presented. In the initial state, in the time region $t_{{\text{w}}} < 2800{\text{ s}}$ (region (I) in Fig. 3a–c), ${\upalpha }$ and $n$ have almost constant values, ${\upalpha } = 1$ and $n \approx 2$. These values indicate that the particles move in a Brownian manner in a simple liquid. In this region, ${\Gamma }$ decreases monotonically, which means an increase in viscosity. In the time region $2800{\text{ s}} < t_{{\text{w}}} < 3400{\text{ s}}$ [region (II)], all the three parameters change drastically. ${\Gamma }$ decreases more rapidly than in the previous time region, ${\upalpha }$ increases from 1 to 2, and $n$ decreases from 2 to approximately 0.8. These behaviors indicate that the particle motion rapidly deviates from the simple Brownian motion in a simple liquid. Thus, the resin is no longer a simple liquid in this region. In the time region $t_{{\text{w}}} > 3400{\text{ s}}$ [region (III)], the change in all the parameters is gradual. ${\Gamma }$ decreases gradually, and ${\upalpha }$ and $n$ are almost constant, ${\upalpha } \approx 2$ and $n \approx 0.8 - 1.0$. These behaviors imply that the dynamics no longer change significantly since the main chemical reactions have settled down. From these results, we can clearly divide the dynamics of the 100 °C curing process into the three time regions, (I) $t_{{\text{w}}} < 2800{\text{ s}}$, (II) $2800{\text{ s}} < t_{{\text{w}}} < 3400,{ }$ and (III) $t_{{\text{w}}} > 3400{\text{ s}},$ from the time dependence of ${\Gamma }$, ${\upalpha }$, and $n$.

Subsequently, the time dependence of the parameters in the 150 °C curing process is presented in Fig. 3d–f. Although ${\upalpha } \approx 1$ and $n \approx 2$ in the initial state of $t_{{\text{w}}} < 1025{\text{ s}}$ [region (A) in Fig. 3d–f] and ${\upalpha } \approx 2$ and $n \approx 0.8 - 1$ in the final state of $t_{{\text{w}}} > 5607{\text{ s}}$ [region (C)] are similar to those of the 100 °C curing process, their behavior differs significantly. First, the time dependence of ${\Gamma }$ does not show a clear boundary in contrast to that in the 100 °C curing process. Particularly, throughout the 150 °C curing process, ${\Gamma }$ decreases smoothly except for a discontinuous jump around $t_{{\text{w}}} \approx$ 1240 s. Second, the behavior of ${\upalpha }$ and $n$ is more scattered than in the 100 °C curing process. During the discontinuous jump of ${\Gamma }$ at $t_{{\text{w}}} \approx$ 1240 s and its quick return to the original line ($1240{\text{ s}} < t_{{\text{w}}} < 1420{\text{ s}}$), ${\upalpha }$ fluctuates between 0.5 and 1.6 (blue marks in Fig. 3d,e). These fluctuations of ${\Gamma }$ and ${\upalpha }$ indicate the coexistence of the fast and slow as well as simple Brownian and non-simple Brownian dynamics. A similar trend, in which ${\upalpha }$ deviates significantly from 1, has been reported in dynamic light scattering measurements for probe diffusion in a gelation process^48^. In addition, the behavior of ${\upalpha }$ in the region $1520{\text{ s}} < t_{{\text{w}}} < 2080{\text{ s}}$ can also be explained by the coexistence of multiple dynamics (green marks in Fig. 3e). In this time range, ${\upalpha }$ is smaller than 1, which means that $g_{2}$ behaves as a stretched exponential function, as shown in $g_{2}$ at 1804s in Fig. 2b, and such a stretched exponential can be expressed by the sum of the multiple relaxations. Furthermore, ${\upalpha }$ increases monotonically from 0.24 to approximately 1. It can be assumed that the multiple dynamic modes converge to a single mode. In the region $2080{\text{ s}} < t_{{\text{w}}} < 4000{\text{ s}}$, ${\upalpha }$ is almost constant. However, the dynamics do not reach a stable state because *n* is fairly scattered in this region and ${\upalpha }$ increases thereafter. This time region ($2080{\text{ s}} < t_{{\text{w}}} < 4000{\text{ s}}$) and the following one ($4000{\text{ s}} < t_{{\text{w}}} < 5607{\text{ s}}$) can be considered the crossover region where ${\upalpha }$ increases from 0.24 to 1.8. From these data, we can conclude that the multiple dynamics coexist in most of the time region $t_{{\text{w}}} < 5700{\text{ s}}$.

**Figure 4:**
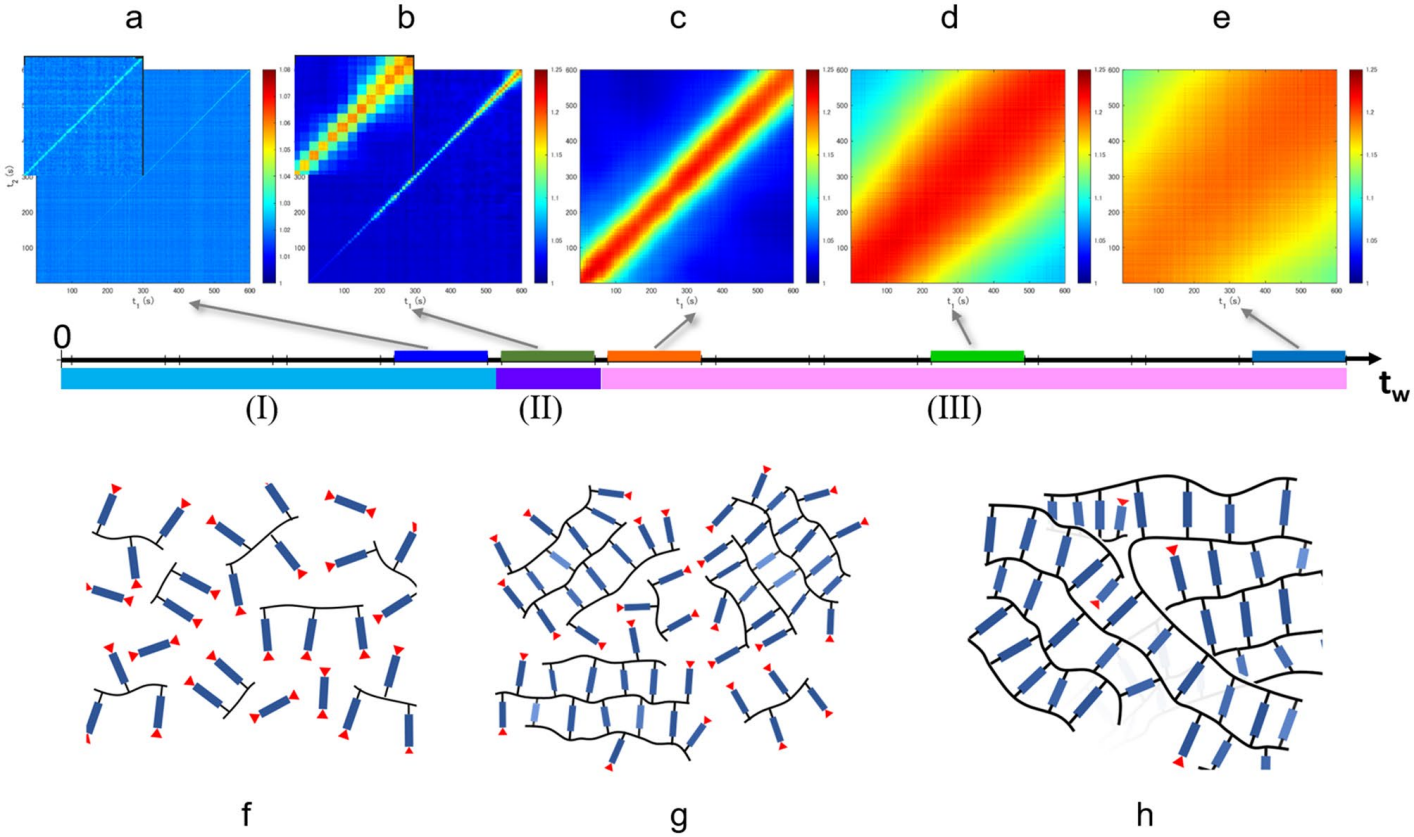
Two-time correlation function $C_{I}$ at *q* = 0.0325 nm^−1^ in the 100 °C curing process at $t_{{\text{w}}}$ = 1448–2048s (**a**), 2136–2736 s (**b**), 2825–3425 s (**c**), 4895–5495 s (**d**), and 7648–8248 s (**e**). The insets in (**a**) and (**b**) show the magnified plot at the last 100 s. The time range of the data is shown with color bars on the $t_{{\text{w}}}$ axis; their colors correspond to the colors of marks in Fig. 3a–c. The schematic illustration of the structure of the epoxy resin in regions (I), (II), and (III) is shown in (**f**), (**g**), and (**h**), respectively. The rod part represents the main chains of BADGE, and the red triangle represents the unreacted end group. The two-time correlation functions were calculated by our programs written in MATLAB 2020b (https://www.mathworks.com). The illustrations were made using Microsoft PowerPoint 2013.

So far, we have discussed the correlation functions $g_{2}$ using Eq. (1), which reflects the averaged dynamics of interest over a period of time. Although the analysis using $g_{2}$ has the advantage that the details of the particle motion can be compared with the parameters obtained from Eq. (2), it hardly expresses the temporal fluctuation of the dynamics. Here, we discuss the temporal variation of the correlation function via a two-time correlation function

3 $$C_{I} \left( {q,t_{1} ,t_{2} } \right) = \frac{{\left\langle {I_{p} \left( {q,t_{1} } \right)I_{p} \left( {q,t_{2} } \right)} \right\rangle_{{\Psi }} }}{{\left\langle {I_{p} \left( {q,t_{1} } \right)} \right\rangle_{{\Psi }} \left\langle {I_{p} \left( {q,t_{2} } \right)} \right\rangle_{{\Psi }} }},$$

where $\left\langle \cdot \right\rangle_{{\Psi }}$ denotes the average over pixels within $q \pm {\Delta }q$^49,50^. From $C_{I}$, the temporal fluctuation of the relaxation time can be represented visually. For instance, in the equilibrium liquid, the width of the diagonal band is constant, thereby indicating that the relaxation time is constant; thus, the dynamics are homogeneous (see Supplementary Fig. 2). In this study, the measurement of 4 ms $\times$ 150,000 frames was repeated 12 times during the curing process, thereby 12 $C_{I}$ were obtained from a set of curing processes (see all the obtained $C_{I}$ in Supplementary Fig. 3 except for the data of which the relaxation time is shorter than the detectable time range). Figure 4a–e demonstrates the representative $C_{I}$ in the 100 °C curing process. In Fig. 4a, $C_{I}$ in the range $2136{\text{ s}} < t_{{\text{w}}} < 2736{\text{ s}}$ [time region (I)] indicates the gradual elongation of the relaxation time, namely the gradual slowing down of the dynamics. In Fig. 4b, for $C_{I}$ in the range $2916{\text{ s}} < t_{{\text{w}}} < 3516{\text{ s}}$ [time region (II)], the relaxation time becomes longer with increasing $t_{{\text{w}}} ,$ which is similar to that in Fig. 4a, but the changes are not gradual but intermittent, thereby indicating a discontinuous dynamic change in this time region. In Fig. 4c–e, $C_{I}$ in time region (III) suggests the disappearance of the intermittent changes and a gradual slowing down. Thus, in the 100 °C curing process, intermittent fluctuations were observed solely in time region (II), and smooth slowdown was observed in time regions (I) and (III).

**Figure 5:**
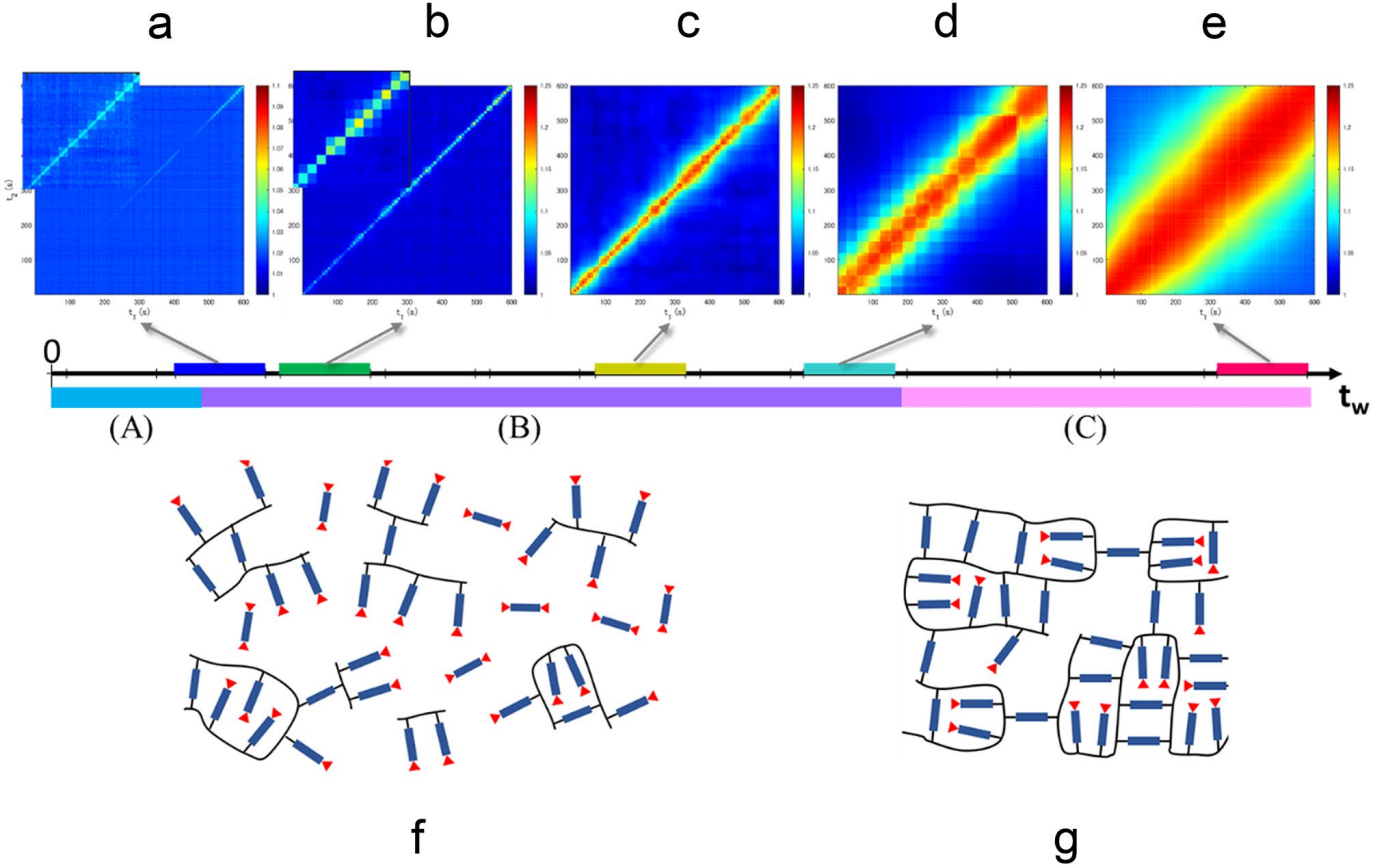
Two-time correlation function $C_{I}$ at *q* = 0.0325 nm^-1^ in the 150 °C curing process at $t_{{\text{w}}}$ = 825–1425 s (**a**), 1524–2124 s (**b**), 3620–4220 s (**c**), 5007–5607 s (**d**), and 7762–8362 s (**e**). The time range of the data is shown with color bars on the $t_{{\text{w}}}$ axis; their colors correspond to the colors of marks in Fig. 3d–f. The schematic illustration of the structure of the epoxy resin in regions (A) and (B) is shown in (**f**), and the illustration in region (C) is shown in (**g**). The two-time correlation functions were calculated by our programs written in MATLAB 2020b (https://www.mathworks.com). The illustrations were made using Microsoft PowerPoint 2013.

In the 150 °C curing process, the intermittent fluctuations were observed over a wider time range from the initial state, in $t_{{\text{w}}} < 5607{\text{ s}}$, compared with the 100 °C curing process, as shown in Fig. 5a–d (see all the obtained $C_{I}$ in Supplementary Fig. 4 except for the data of which the relaxation time is shorter than the detectable time range). These intermittent fluctuations indicate the coexistence of fast and slow dynamics, which originates from the heterogeneity of the matrix due to the simultaneous reaction of the oligomerization, polymerization, and cross-linking reaction from the initial stage in the 150 °C curing process. In the time region $t_{{\text{w}}} > 5607{\text{ s}}$ [region (C)], where the curing progressed sufficiently and ${\upalpha }$ and *n* were stable (seen in Fig. 3e,f), no intermittent fluctuations were observed, as shown in Fig. 5e.

After approximately 5500 s of these measurements, parameters ${\Gamma }$, ${\upalpha }$, and *n* are almost constant at both 100 °C and 150 °C, as shown in Fig. 3. Fluctuations in dynamics are observed in $C_{I}$, and such temporal fluctuations can include important information for the curing mechanism. The fluctuation of $C_{I}$ can be discussed quantitatively by its normalized variance^2^

4 $${\upchi }\left( {q,t} \right) = \frac{{\left\langle {C_{I}^{2} \left( {q,t_{1} ,t} \right)} \right\rangle_{{t_{1} }} - \left\langle {C_{I} \left( {q,t_{1} ,t} \right)} \right\rangle_{{t_{1} }}^{2} }}{{\left\langle {C_{I} \left( {q,t_{1} ,t = 0} \right)} \right\rangle_{{t_{1} }}^{2} }}.$$

The parameter ${\upchi }$ exhibits a peak around the inflection point of $g_{2} \left( {q,t} \right)$, and the height of this peak is proportional to the variance of the characteristic relaxation time. The experimentally measured variance ${\upchi }\left( {q,t} \right)$ is affected by statistical noise owing to the use of a finite number of pixels $n_{{\text{p}}}$. Thus, we corrected the measured ${\upchi }\left( {q,t} \right)$ by applying a correction procedure based on extrapolation, $1/n_{{\text{p}}} = 0$ as reported previously^51,52^.

**Figure 6:**
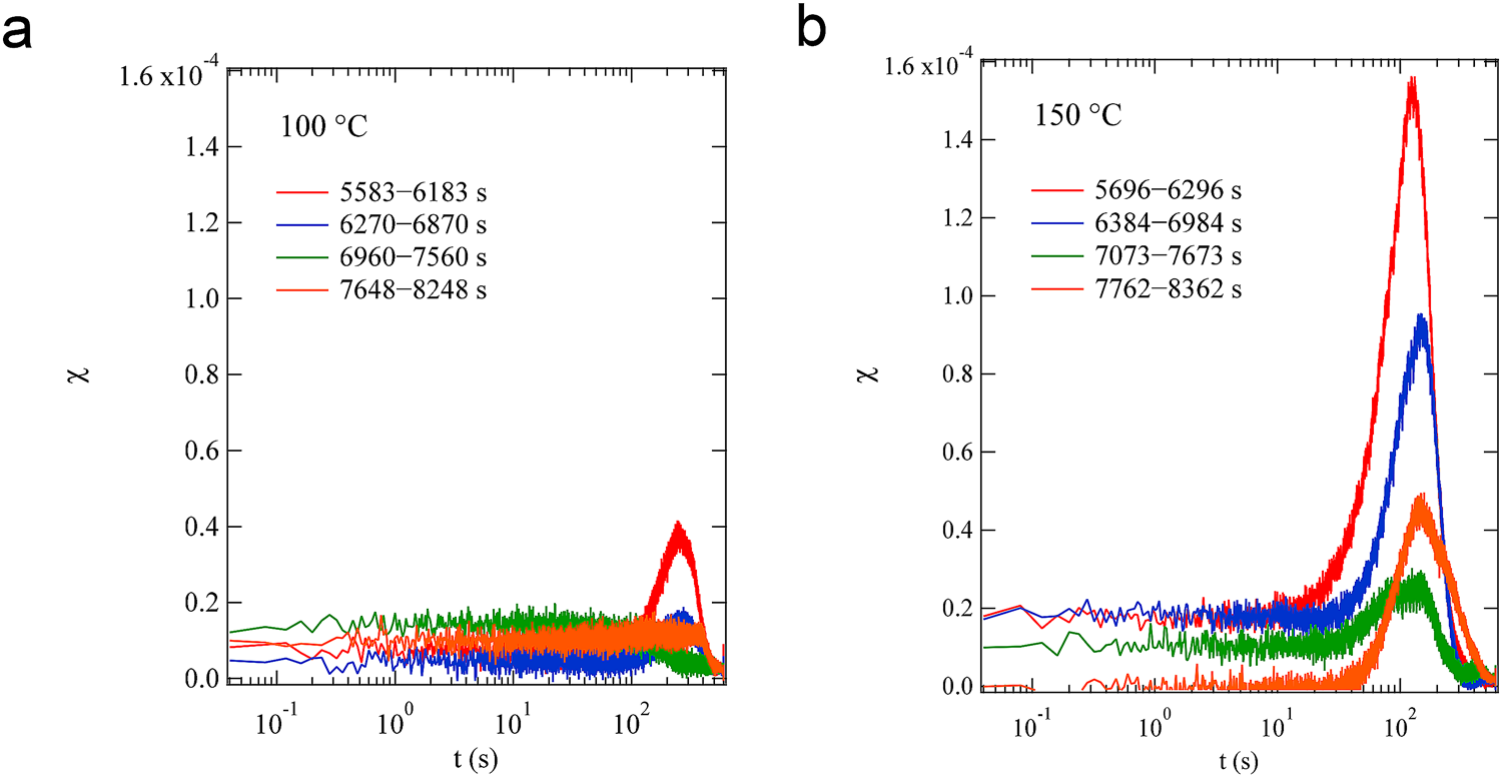
${\upchi }$, the fluctuation of $C_{I}$ calculated from Eq. (4), at *q* = 0.0325 nm^−1^ obtained from the four measurement sets at $t_{{\text{w}}} > 5583{\text{ s}}$ in the 100 °C curing process (**a**) and at $t_{{\text{w}}} > 5696{\text{ s}}$ in the 150 °C curing process (**b**).

Figure 6a,b show ${\upchi }$ that at *q* = 0.0325 nm^−1^ obtained from the last four measurement is in the range $t_{{\text{w}}} > 5583$ s in the 100 °C curing process and in $t_{{\text{w}}} > 5696$ s in the 150 °C curing process, respectively. For the 100 °C curing process, ${\upchi }$ obtained from $5583{\text{ s}} < t_{{\text{w}}} < 6183{\text{ s}}$ shows a clear peak (red line), which represents the instability of dynamics. However, the peak height decreases with increasing $t_{{\text{w}}}$, and no clear peak is observed for the last measurement in the range $7648{\text{ s}} < t_{{\text{w}}} < 8248{\text{ s}}$ (orange line). These peak behaviors indicate that the dynamics become stable within the measurement time in the 100 °C curing process. In the 150 °C curing process, all the four data show high peaks, although the curing reaction is faster than that in the 100 °C curing process and a long time has passed since the gelation point obtained by the macroscopic rheological measurement (see Supplementary Fig. 1). Moreover, the peak height does not decrease monotonically; ${\upchi }$ in $7762{\text{ s}} < t_{{\text{w}}} < 8362{\text{ s}}$ has a higher peak (orange line) than ${\upchi }$ in $7073{\text{ s}} < t_{{\text{w}}} < 7673{\text{ s}}$ (green line). This indicated that the dynamics were still unstable in that time region, and the dynamical heterogeneity of the resin cured at 150 °C was more remarkable than that of the resin cured at 100 °C. Similar results were obtained in all the measured *q* ranges (see Supplementary Fig. 5).

### Cross-link inhomogeneity of cured resins

In addition to the investigations of the dynamics of the curing process presented so far, we also investigated the cross-link inhomogeneity of the cured materials. Using ^1^H-pulse NMR technique, we investigated the microscopic structures of the cured resins obtained from the two different curing processes, sample A (100 °C for 2 h + 150 °C for 5 h) and sample B (150 °C for 5 h).

**Figure 7:**
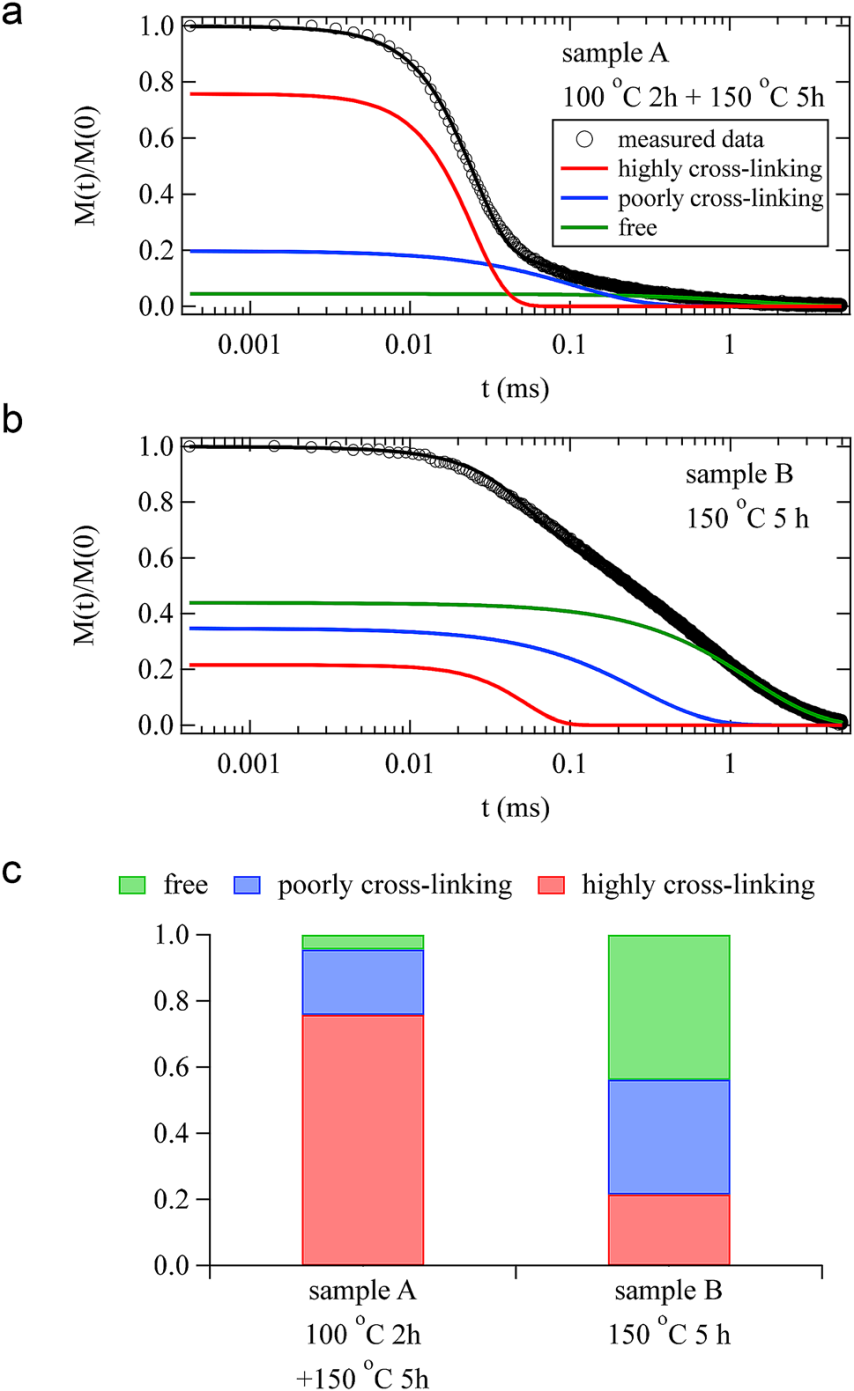
^1^H-pulse nuclear magnetic resonance (NMR) spectrum for sample A (the solid epoxy resin cured at 100 °C for 2 h and 150 °C for 5 h) (**a**). ^1^H-pulse NMR spectrum for sample B (the solid epoxy resin cured at 150 °C for 5 h) (**b**). Both measurements were carried out at 120 °C. The solid lines in (**a**) and (**b**) are obtained from the fitting analysis with Eq. (6). (**c**) The molar fraction of protons for the hard polymer in the highly cross-linking part, the soft polymer in the poorly cross-linking part, and the free polymer obtained from the fitting analysis with Eq. (6).

The ^1^H-pulse NMR measurements were carried out at 120 °C, which is higher than the glass transition temperature (66 °C for sample A and 48 °C for sample B) obtained from dynamic mechanical analysis (DMA) measurements shown in Supplementary Fig. 6. Figure 7a,b present the results for the ^1^H-pulse NMR measurements of samples A and B, respectively. The spectrum for sample B shows a longer relaxation time than sample A. In general, the spin–spin relaxation function can be expressed by

5 $$\frac{M\left( t \right)}{{M\left( 0 \right)}} = \exp \left[ { - \frac{1}{a}\left( {\frac{t}{{T_{2} }}} \right)^{a} } \right],$$

where $M\left( t \right)/M\left( 0 \right)$, $a$, and $T_{2}$ denote the normalized magnetization intensity at $t$, exponent of the decay function, and time constant representing the spin–spin relaxation, respectively^45,46^. The range of $a$ is from 1, for an exponential decay, to 2, for a Gaussian decay. In polymers having low molecular mobility, such as in the crystalline and glassy states, the relaxation typically follows the Gaussian decay. In polymers with greater molecular mobility, such as in the liquid and rubbery states, the relaxation follows an exponential slow decay. The cured materials should be composed of a hard polymer with low molecular mobility due to the high cross-linking and a soft polymer with great molecular mobility due to the poor cross-linking or free state. In fact, the measured data were well described by the following function:

6 $$\frac{M\left( t \right)}{{M\left( 0 \right)}} = f_{{{\text{Hard}}}} \exp \left[ { - \frac{1}{2}\left( { \frac{t}{{T_{{2,{\text{Hard}}}} }}} \right)^{2} } \right] + f_{{{\text{Soft}}1}} \exp \left( { - \frac{t}{{T_{{2,{\text{Soft}}1}} }}} \right) + f_{{{\text{Soft}}2}} \exp \left( { - \frac{t}{{T_{{2,{\text{Soft}}2}} }}} \right),$$

where $f_{{{\text{Hard}}}}$, $f_{{{\text{Soft}}1}}$, and $f_{{{\text{Soft}}2}}$ represent the molar fraction of protons for the hard polymer in the highly cross-linked part, the soft polymer in the poorly cross-linked part, and the free polymer, respectively. $T_{{2,{\text{Hard}}}}$, $T_{{2,{\text{Soft}}1}}$, and $T_{{2,{\text{Soft}}2}}$ are the relaxation times of the respective polymers with the relation $T_{{2,{\text{Soft}}2}} > T_{{2,{\text{Soft}}1}} > T_{{2,{\text{Hard}}}}$. Through the fitting analysis with Eq. (6), the molar fractions were obtained as shown in Fig. 7c. For sample A, the proportions of the high cross-linking component, low cross-linking component, and free polymer component are 75.8, 19.8, and 4.4%, respectively, whereas for sample B, they are 21.5, 34.7, and 43.8%, respectively. These results clearly indicate that sample A is more highly cross-linked and contains fewer free polymers than those in sample B.

## Discussion

The dynamic studies of the curing process using XPCS revealed that the microscopic dynamics were quite different according to the curing temperature.

In the 100 °C process, the kinetics were clearly distinguished into three different regions (I)–(III). In region (I), the probe nanoparticles showed Brownian motion in a simple liquid, thereby indicating that the epoxy resin was in a liquid state. The increase in viscosity was considered to indicate the progress of oligomerization of BADGE, as shown in Fig. 4f. In the BADGE/C11Z-CN system, epoxy groups are linked by a catalytic reaction to form a main chain, and the aromatic ring hangs as a side chain to generate an oligomer. The dynamics slow down as the viscosity increases owing to the increase in molecular weight. In region (II), it was considered that the cross-linking reaction between oligomers proceeded to form a network structure, as shown Fig. 4g. In this process, the motion of the nanoparticles is no longer simple Brownian, and a drastic increase in viscosity drastically slows down the dynamics. Furthermore, because a large viscosity difference is generated between the network structure formed and unformed parts, the dynamics of the nanoparticles show heterogeneity. This heterogeneity is reflected as an intermittent change, as shown in Fig. 4b. In region (III), the cross-linking reaction, as shown in Fig. 4h, is almost complete and a network structure is formed; thus, the epoxy resin is in a solid state. The Fourier transform infrared (FTIR) spectroscopy measurement also shows that the main reaction was completed around 3000 s (shown in Supplementary Fig. 7a); thereafter, the reaction of the remaining epoxy groups proceeded slowly. In this process, the dynamics gradually slow down. Moreover, because the network structure is almost formed, no intermittent fluctuations are shown in $C_{I}$. As described, it was revealed that (I) oligomerization in a liquid state, (II) gelation, and (III) residual cross-linking reaction proceeded in order in the 100 °C curing process. This ordered process resulted in cured materials with low dynamical heterogeneity and a dense network, as shown by ^1^H-pulse NMR measurements.

In the 150 °C curing process, the parameter values of ${\upalpha } \approx 1$ and $n \approx 2$ in the initial stage and ${\upalpha } \approx 2$ and $n \approx 1$ in the final stage were similar to the respective values in the 100 °C curing process, as shown in Fig. 3. However, there was a significant difference: there was not clear regional division in the 150 °C curing process in contrast to the 100 °C curing process. The differences in dynamic behavior between the two processes are also shown in $C_{I}$. In all the processes before solidification [regions (A) and (B) in Fig. 3d–f], $C_{I}$ had intermittent behavior, as shown in Fig. 5a–d, whereas such intermittent behavior was observed solely in region (II) in the 100 °C curing process. This indicates that the domains with fast and slow dynamics coexisted over a wide $t_{{\text{w}}}$ range from the initial process to solidification; thus, the oligomerization and the cross-linking reaction proceeded simultaneously, as schematically shown in Fig. 5f. During the reaction, a large number of unreacted epoxy groups are generated. FTIR spectroscopy results showed that the reaction almost stopped at a degree of cure approximately 0.4, as shown in Supplementary Fig. 7b. Consequently, a structure with low cross-link density was formed, as schematically shown in Fig. 5g, which was also indicated in the ^1^H-pulse NMR results; this structure caused high dynamical heterogeneity.

Here, we discuss the difference of the curing mechanism between the 100 and 150 °C curing processes. In the present epoxy system, the curing reaction proceeded by the chemical reaction between the epoxy groups at the ends of BADGE, which was promoted by the catalyst C11ZCN. The energy barrier of the first bonding reaction of the epoxy group of BADGE should be lower than that of the second reaction after becoming part of the oligomer. The difference of the energy barrier could affect the curing kinetics between the 100 and 150 °C processes. In the 100 °C process, in the early stage, linear oligomers were formed by the reaction of the first epoxy group, and, in the subsequent stage, the cross-linking reaction by the reaction of the second epoxy group proceeded. On the other hand, in the 150 °C curing process, the second bonding reaction as well as the first reaction occurred in the early stage because of the high temperature. Those reactions generated the coarse cross-linking networks in the early stage and caused the transition from chemical control kinetics to those of diffusion control. In the 150 °C process, as shown in the result of the rheological measurements (Supplementary Fig. 1b), the macroscopic rheological values showed solidification at about 800 s, indicating that the molecular diffusion was greatly reduced. This solidification time agrees with the time taken by the reaction of the epoxy group to settle down in the FTIR measurement (Supplementary Fig. 7b). Those reductions of molecular diffusion suppressed the degree of cure around 0.4 in the 150 °C curing process, as shown in the FTIR results, whereas the degree of cure progressed to 0.8 in the 100 °C curing process.

In summary, XPCS measurements during the curing process at two different temperatures revealed the overall dynamics of the catalytic epoxy resin from liquid to solid state. Furthermore, it was shown that there was a large difference in the dynamical heterogeneity in the solid state. By interpreting the XPCS results through comparison with various measurement results, a concrete overview of the network structure was presented. In this study, we investigated the curing process of the catalytic epoxy resin. However, there are various types of epoxy resins, and their curing mechanisms vary accordingly. Future studies are needed to investigate the dynamics of these various epoxy resins to provide a more general insight. Moreover, although we used the nanoparticles having diameters of 120 nm in the present study, it has been reported in particle-tracking measurements of the curing process of an epoxy-amine mixture that the particle size can affect particle movement^53^. Thus, it will be interesting to analyze the size dependence of probe particles. These results will be particularly useful for improving the quality of thermosetting epoxy resins as well as various materials, such as 3D printing products^54,55^.

## Materials and methods

### Materials

The chemical structures of epoxy resins in this study are shown in Fig. 1. We used bisphenol A diglycidyl ether (BADGE) (Mitsubishi Chemical, jER828) as the base resin and 1-(2-cyanoethyl)-2-undecylimidazole (C11ZCN) (SHIKOKU CHEMICALS CORPORATION) as the catalyst. BADGE and C11ZCN were mixed in a weight ratio of 100:3.

For the cured resins used in the DMA and ^1^H-pulse NMR experiments, two different types of epoxy materials were prepared: the epoxy raw resin mixture was degassed under reduced pressure, it was poured into a mold with a thickness of 1 mm and cured at 100 °C for 2 h and 150 °C for 5 h or 150 °C for 5 h.

In the sample used in the XPCS measurements, silica particles were homogeneously distributed in a mixed solution of the main agent and catalyst, which was utilized as a probe. Particularly, silica particles with a diameter of 120 nm (Nissan Chemicals, Japan) were dispersed in an epoxy resin (BADGE/C11ZCN = 100/3 (g/g)) at ~ 1 vol%. By applying small-angle X-ray scattering, we confirmed that the silica particles were homogeneously dispersed and the interparticle interaction was negligible within the measured q range (see Supplementary Fig. 8).

### XPCS measurements

The XPCS measurements were performed at beam line BL03XU at SPring-8 (Hyogo, Japan)^56^. The undulator source and Si (111) monochromator were tuned to an energy of 8.00 keV. The sample was irradiated with partially coherent X-rays obtained by passing the beam through a pinhole of 20 µm diameter, and the scattered X-rays were detected using an EIGER 1 M two-dimensional detector (Dectris, Switzerland) mounted approximately 8 m at the back of the sample.

In the XPCS measurements during the curing process, we analyzed the fluctuation of the speckle patterns scattered from the dispersed tracer particles in the epoxy resins. The samples in the liquid state were enclosed in cells designed to have homogeneous temperature using aluminum foil as window materials, placed in a heat bath at 100 or 150 °C, and the change in dynamics during the curing process was measured^57^.

### ^1^H-pulse NMR spectroscopy

^1^H-pulse NMR experiments were performed using a Bruker Minispec mq20 at a proton resonance frequency of 20 MHz. The signals were acquired using the solid-echo pulse sequence with a ${\uppi }/2$ pulse duration of 3.1 µs, dead time of 8.3 µs, dwell time of 1 µs, recycle delay of 1 s, and 64 transients. The epoxy cured product crushed to a size between 1 and 2 mm was filled in a glass tube Bruker E1405321_10 with a diameter of 10 mm, and its temperature was maintained at 120 °C during the measurement.

## Supplementary Information

Supplementary Information.

## Data Availability

The raw data generated and analyzed as a part of this study are available from the corresponding author upon request.
