# Supplementary material for: Heterogeneous dynamics in the curing process of epoxy resins
Source: Sci Rep. 2021 May 17;11:9767. doi: 10.1038/s41598-021-89155-x (PMC8129072; doi:10.1038/s41598-021-89155-x)
Supplement: Supplementary file 1 — Supplementary Information. [file 41598_2021_89155_MOESM1_ESM.docx]

**Heterogeneous dynamics in the curing process of epoxy resins**

Taiki Hoshino, Yasushi Okamoto, Atsushi Yamamoto, Hiroyasu Masunaga

**Supplementary information**

**Rheometry**

It is well known that the mechanical properties of the thermoset epoxy resin change during the curing process; particularly near the gel point, the storage elastic modulus *G'* and the loss elastic modulus *G''* change drastically. In this study, the elastic modulus during the curing process was investigated via rheometry. The viscoelastic properties were measured using a Thermo Scientific HAAKE MARS 60 rheometer with the parallel plate rheometry method. The sample was subjected to sinusoidal deformations at an amplitude of 0.1% and a frequency of 1 Hz. The sample was placed at room temperature, and the temperature was elevated. After reaching the target temperature, rheometric measurements were started.

The elastic moduli obtained by rheometry at 100 °C and 150 °C are shown in Supplementary Fig. 1**a** and 1**b**, respectively. *G'* and *G''* show an increase at around 2300 s in the 100 °C curing process and at around 600 s in the 150 °C curing process. This means that the curing reaction at 150 °C proceeded much faster than at 100 °C.


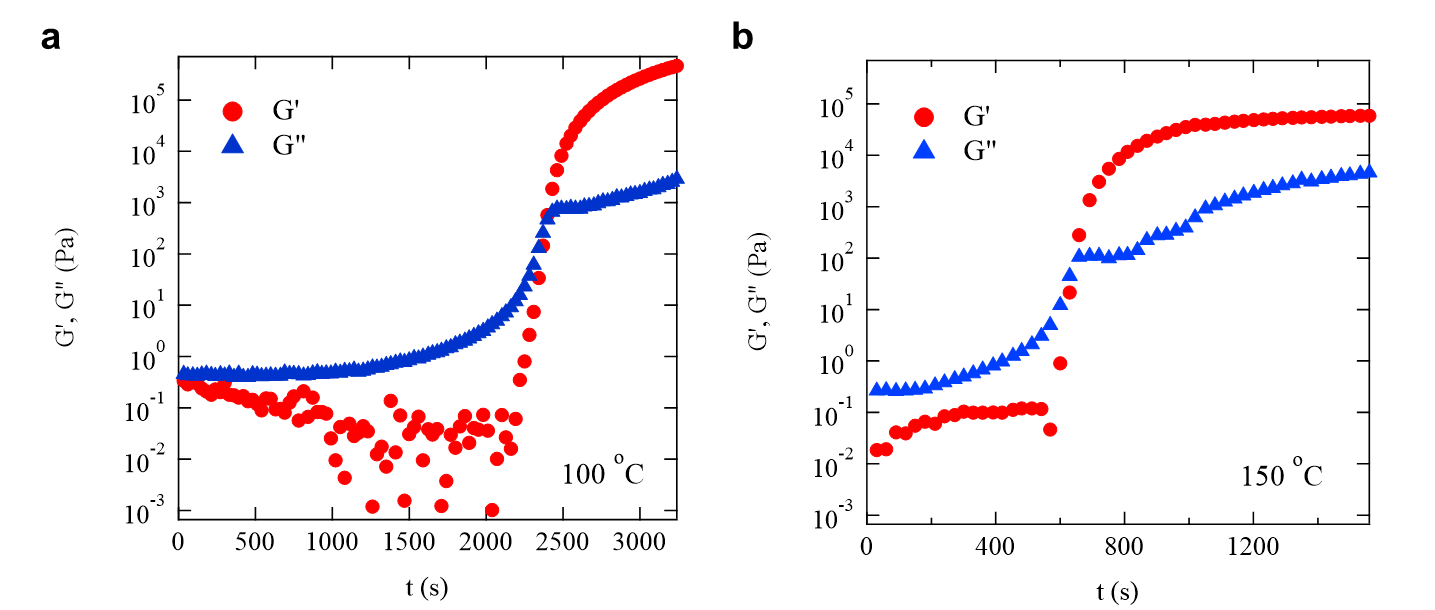


**Supplementary Fig. 1:** Time dependence of the storage elastic modulus $G'$ and the loss elastic modulus $G''$ obtained from rheometry during curing process at 100 °C (**a**) and 150 °C (**b**).

**Two-time correlation function in the equilibrium liquid state**

As an example of the two-time correlation function $C_{I}$ in the equilibrium liquid state at 60 °C, $C_{I}$ at *q* = 0.0325 nm^-1^ is shown in Supplementary Fig. 2, which was obtained from 500 frames with an exposure time of 1 ms. The width of the diagonal band (around $C_{I}>1.1)$ was constant, indicating that the relaxation time was constant.


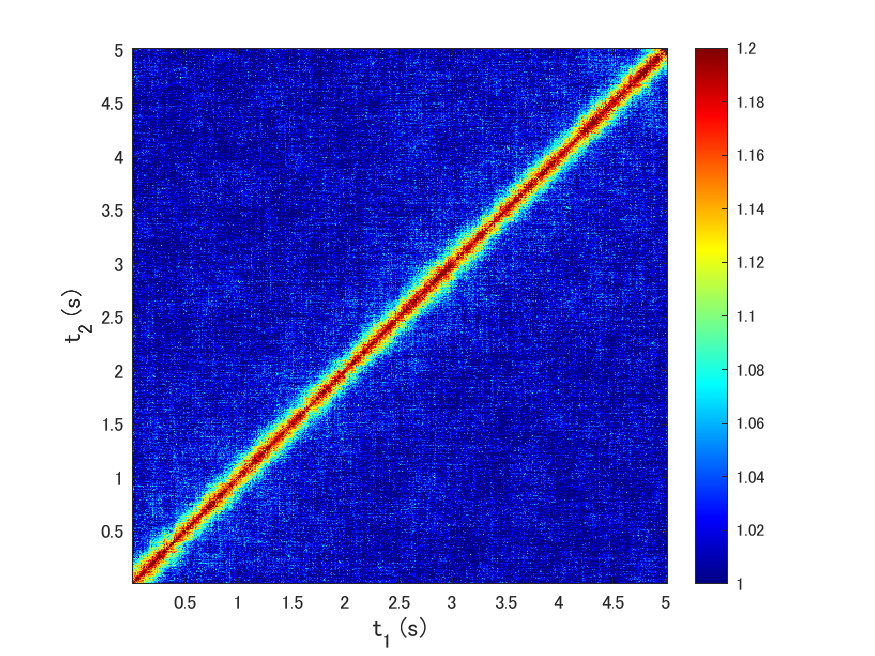


**Supplementary Fig. 2:** Two-time correlation function $C_{I}$ in the equilibrium liquid state at 60 °C. The two-time correlation functions were calculated by our programs written in MATLAB 2020b (https://www.mathworks.com).

**Two-time correlation function in the curing process**

In this study, the measurement of 4 ms $\times$ 150 000 frames was repeated 12 times during the curing process; thus, 12 $C_{I}$ profiles were obtained from one curing process. All the obtained $C_{I}$ profiles from the 100 °C curing process and the 150 °C curing process, except for the case where the relaxation time was shorter than the detectable time range, are shown in Supplementary Figs. 3 and 4, respectively.


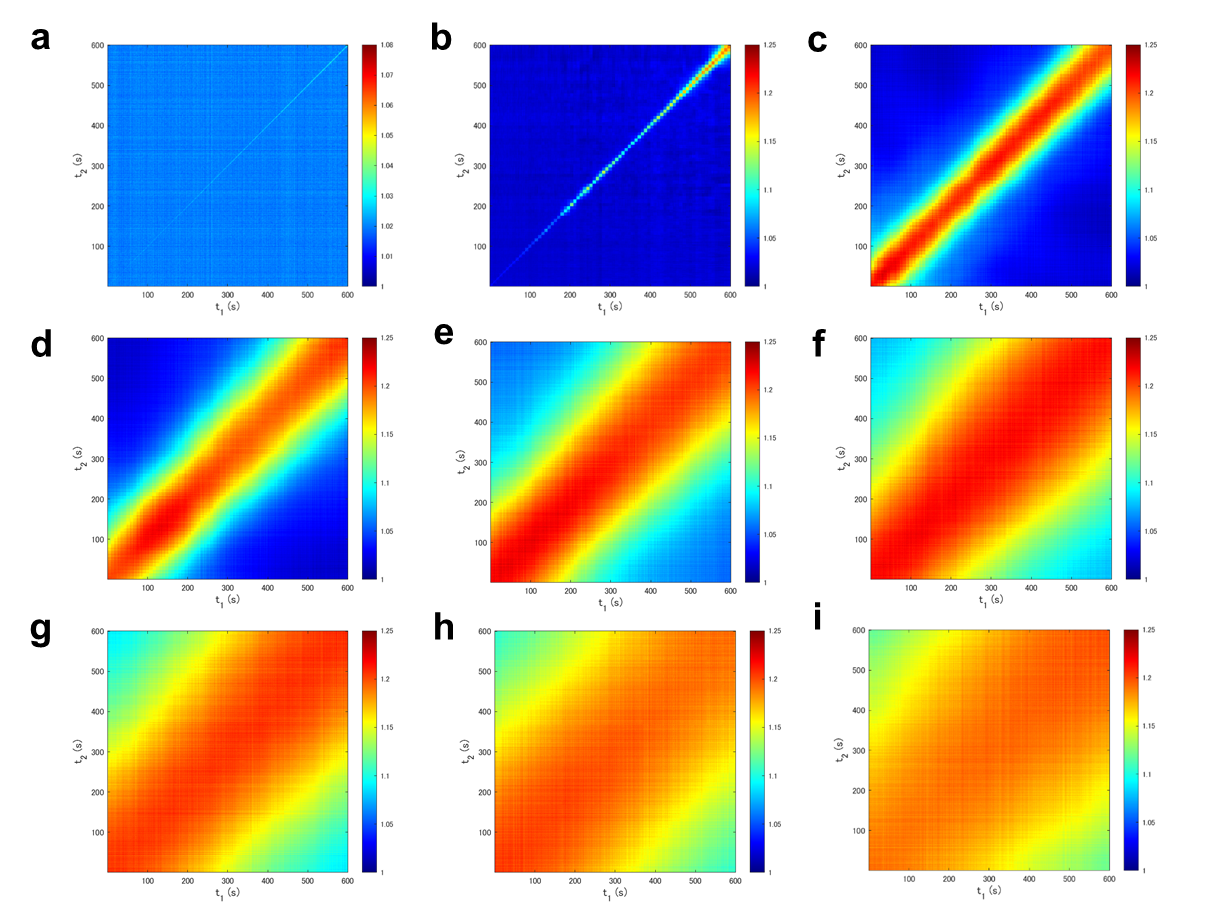


**Supplementary Fig. 3:** $C_{I}$ at $t_{w}$= 2136–2736 s (**a**), 2825–3425 s (**b**), 3508–4108 s (**c**), 4200–4800 s (**d**), 4895–5495 s (**e**), 5583–6183 s (**f**), 6270–6870 s (**g**), 6960–7560 s (**h**), and 7648–8248 s (**i**). $C_{I}$ in the range $t_{w}$< 2136 s in which relaxation time is shorter than the frame time of 4 ms are not shown. The two-time correlation functions were calculated by our programs written in MATLAB 2020b (https://www.mathworks.com).


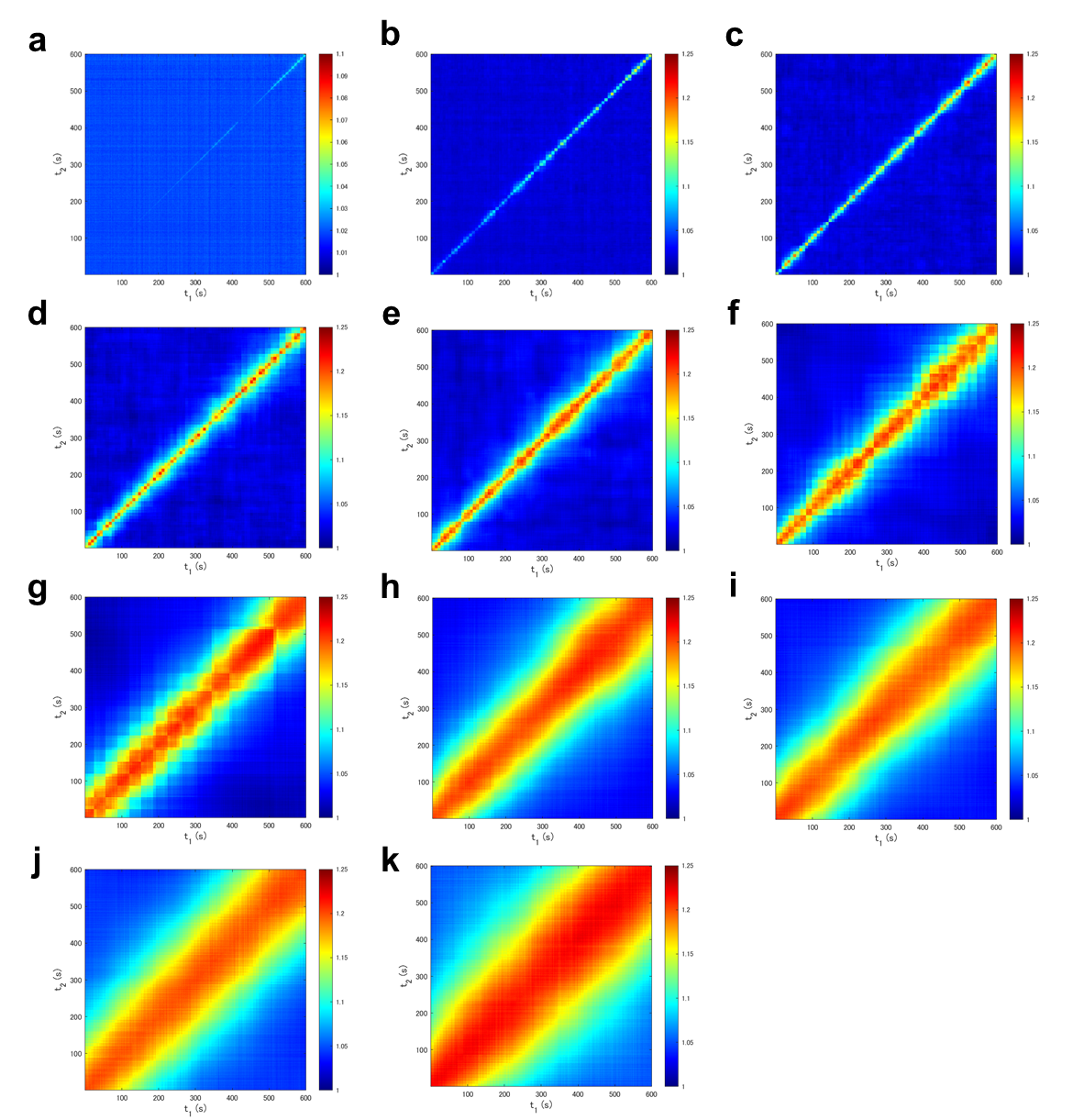


**Supplementary Fig. 4:** Obtained $C_{I}$ at $t_{w}$= 825–1425 s (**a**), 1524–2124 s (**b**), 2222–2822 s (**c**), 2916–3516 s (**d**), 3620–4220 s (**e**), 4320–4920 s (**f**), 5007–5607 s (**g**), 5696–6296 s (**h**), 6384–6984 s (**i**), 7073–7673 s (**j**), 7762–8362 s (**k**). $C_{I}$ in the range $t_{w}$< 825 s in which relaxation times shorter than the frame time of 4 ms are not shown. The two-time correlation functions were calculated by our programs written in MATLAB 2020b (https://www.mathworks.com).

**Peak height and peak position of** $\boldsymbol{\chi}$ **at various *q* after 5500 s**

The temporal fluctuation of $C_{I}$ in the range $t_{w}>5500 s$, $\chi$ at *q* = 0.0325 nm^-1^, is shown in Fig. 6. To exhibit the behavior of $\chi$ at the other *q* ranges, the height of the peak $\chi^{*}$ and the peak position $\tau^{*}$ obtained from the measurement in the range $t_{w}>5500 s$ as well as various *q* are shown in Supplementary Fig. 5. The *q* dependence of $\chi^{*}$ is not clear, owing to the limited scattering intensity since the calculation of $\chi$ requires high statistics. $\tau^{*}$ shows a relatively clear *q* dependence, which decreases with increasing *q*.

In the 100 °C curing process, $\chi^{*}$ is relatively large in the range ${5583<t}_{w}<6183 s$ at all the measured *q* ranges. However, it became smaller after that, and almost no peak was observed in the range $t_{w}>6270 s$. In the 150 °C curing process, $\chi^{*}$ remains large overall, thereby indicating that the temporal fluctuation remains. The indication of the fluctuation is also shown in $\tau^{*}$. In the 150 °C curing process, $\tau^{*}$ does not show monotonic slowdown behavior. In the 100 °C curing process, however, $\tau^{*}$ clearly shows a slowdown with increasing $t_{w}$. These results indicate that the relatively large deviation of dynamics in the 150 °C curing process remains in this time region compared to the 150 °C curing process.


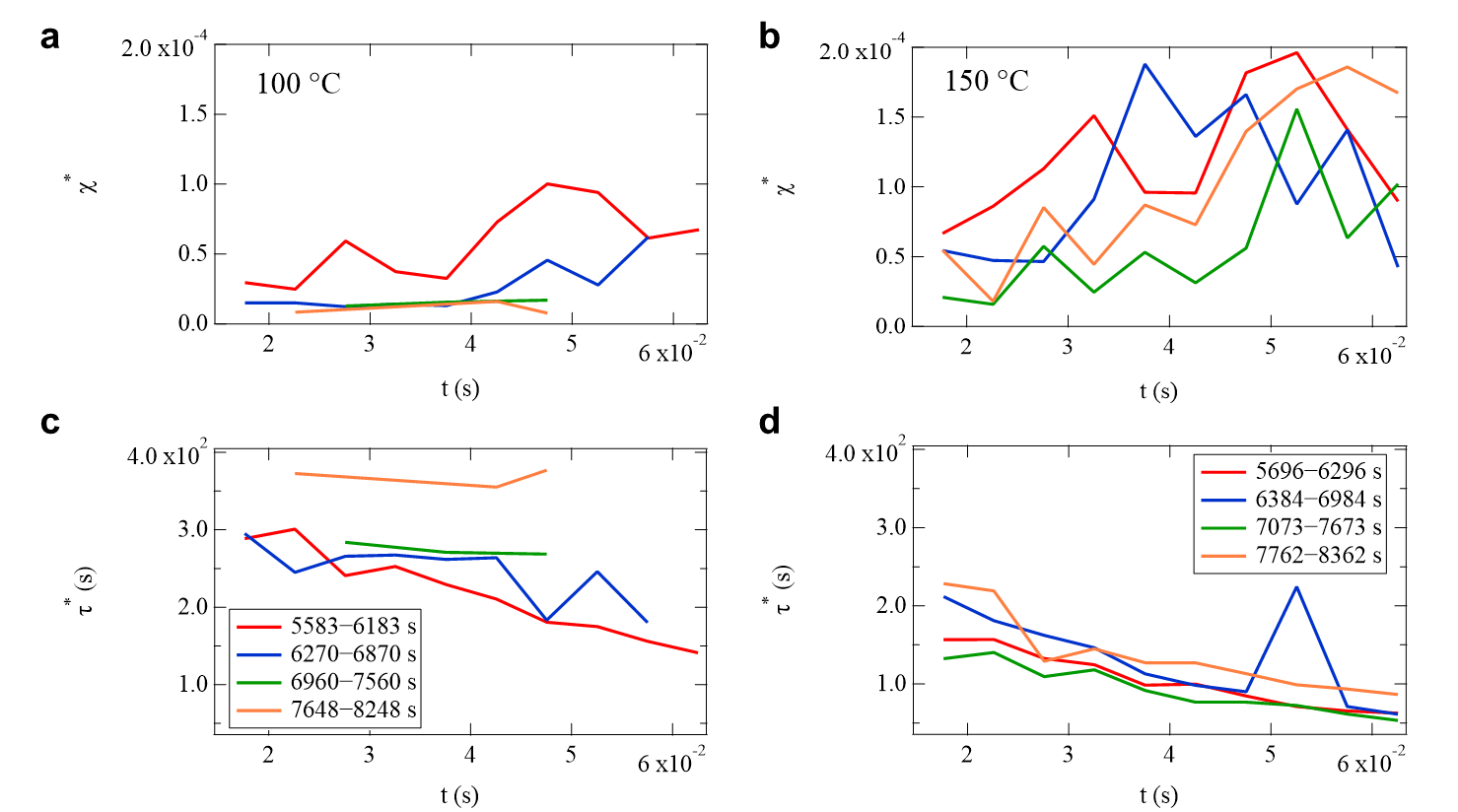


**Supplementary Fig. 5:** *q* dependence of the height of the peak $\chi^{*}$ and the peak position $\tau^{*}$ obtained from the measurement at $t_{w}>5500 s$. **a** and **b** depict $\chi^{*}$ at 100 °C and 150 °C, respectively. **c** and **d** depict $\tau^{*}$ at 100 °C and 150 °C, respectively.

**Dynamic mechanical analysis (DMA)**

The viscoelastic properties of the epoxy-cured materials were analyzed via DMA, performed using a DMS6100 (SII NanoTechnology Inc.). Rectangular specimens of 50$\times$9$\times$1 mm^3^ were used, and the measurement was performed at an oscillation amplitude of 10 $\mu m$ and a frequency of 1 Hz, from 0 °C to 300 °C at a heating rate of 2 °C/min.

The storage elastic modulus $G'$, loss elastic modulus $G''$, and loss coefficient $tan\delta=G^{''}/G'$ obtained by DMA for sample A (100 °C for 2 h + 150 °C for 5 h) and sample B (150 °C for 5 h) are shown in Supplementary Fig. 6 **a**, **b**, and **c**, respectively. In all the measured temperature ranges, $G'$ for sample A is larger than $G'$ for sample B; moreover, $tan\delta$ for the 100 °C cured material has a peak at 66 °C, whereas that for the 150 °C cured material has a peak at 48 °C, thereby indicating that sample A has a much higher glass transition temperature than did sample B. Furthermore, sample B has a higher peak than that of sample A. These results indicate that sample A is more elastic and shows higher stiffness for applications compared to sample B.


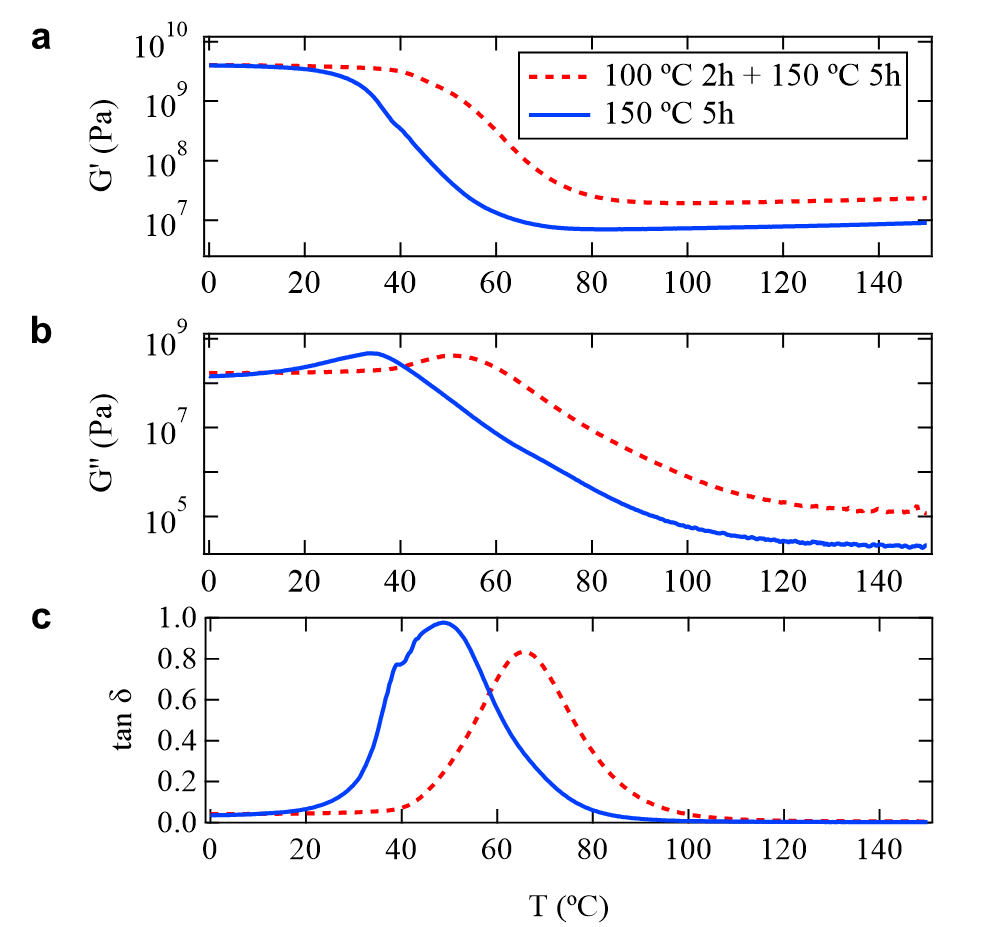


**Supplementary Fig. 6:** Storage elastic modulus $G'$, loss elastic modulus $G''$, and loss coefficient $tan\delta=G^{''}/G'$ obtained by dynamic mechanical analysis for sample A (100 °C for 2 h + 150 °C for 5 h) and sample B (150 °C for 5 h).

**Fourier transform infrared (FTIR) spectroscopy during curing process**

The chemical reaction process was evaluated by FTIR spectroscopy in transmission geometry using an FTS3000, UMA600 from Agilent Technologies (USA). In the measurements, a quartz beam splitter and a mercury cadmium telluride (MCT/A) detector were used, and all the spectral data were measured at 8 cm^-1^ resolution using 512 scans in the spectral range 8000–4000 cm^-1^ with a time resolution of approximately 1 min. The sandwiched epoxy resin between glasses with 1-mm-thick spacer was heated using a temperature-controlled stage (Linkam 10036L) with a heating rate of 100 °C/min and maintained at 100 °C or 150 °C under a nitrogen atmosphere. The measurements were performed with a time resolution of approximately 30 s.

The reaction rate of cross-linking was evaluated from the abundance ratio between the epoxy radical and aromatic ring. The ratio of the absorbance peak $A_{\mathrm{epoxy}}/A_{\mathrm{aroma}}$ was obtained, where $A_{\mathrm{epoxy}}$ and $A_{\mathrm{aroma}}$ are the absorbances at 4530 cm^-1^ and 4620 cm^-1^, respectively. Supplementary Figs. 7**a** and **b** show the temporal changes in the absorbance peak ratio $A_{\mathrm{epoxy}}/A_{\mathrm{aroma}}$ during the curing process at 100 °C and 150 °C, respectively. The reaction rate, which is obtained from the time derivative term $d(A_{\mathrm{epoxy}}/A_{\mathrm{aroma}})/dt$, is also shown. In the 100 °C curing process, the reaction proceeded slowly, and $d(A_{\mathrm{epoxy}}/A_{\mathrm{aroma}})/dt$ started to decrease around 2900 s, which is assumed to be the gel point. Finally, the reaction ratio was approximately 0.8.

In the 150 °C curing process, the reaction progressed rapidly and almost stopped after reaching 0.4 around 1000 s. Only approximately 40% of the epoxy function reacted, which is only half of that in the 100 °C curing process.


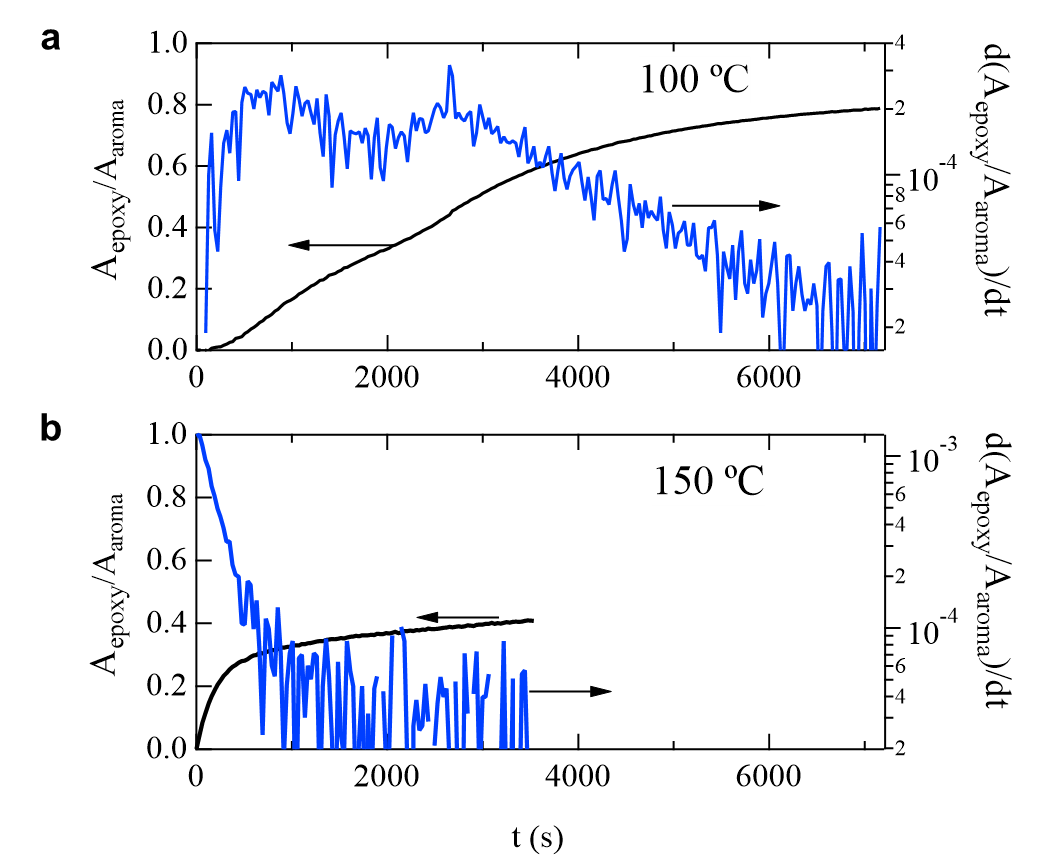


**Supplementary Fig. 7:** Time dependence of the abundance ratio between epoxy radical and aromatic ring $A_{\mathrm{epoxy}}/A_{\mathrm{aroma}}$ (black line) and the reaction rate of $d(A_{\mathrm{epoxy}}/A_{\mathrm{aroma}})/dt$ (blue line) in the 100 °C curing process (**a**) and 150 °C curing process (**b**) obtained from Fourier transform infrared spectroscopy.

**Confirmation of homogeneous dispersibility of the probe particles**

The dispersibility of the probe silica particles (diameter of 120 nm) in an epoxy resin was investigated by small angle X-ray scattering measurements. As shown in Supplementary Fig. 2, the scattering profile was well represented by the form factor of a spherical particle, and the interparticle interaction was negligible within the measured *q* range.

**Supplementary Fig. 8:** Small angle X-ray scattering profile for the silica particles in the epoxy resin. The solid line is a fitting curve obtained using a form factor of a spherical particle.

**Effect of silica nanoparticles**

Although the concentration of dispersed nanoparticles (1 vol%) is much lower than in typical filler applications, it is significant to check the influence of nanoparticles, because it has been reported that even a small amount of dispersion can affect the physical properties. We performed the rheological measurements and the ^1^H-pulse NMR measurements for the samples containing nanoparticles (1 vol%).

The results of the rheological measurements are shown in Supplementary Fig. 9. *G'* and *G''* show an increase at around 2300 s in the 100 °C curing process and at around 500 s in the 150 °C curing process. Those time ranges are very close to the data without nanoparticles shown in Supplementary Fig. 1.

The results of ^1^H-pulse NMR measurements are shown in Supplementary Fig. 10. The spectrum for sample B with nanoparticles shows a much longer relaxation time than sample A with nanoparticles. For sample A with nanoparticles, the proportions of the high cross-linking component, low cross-linking component, and free polymer component are 64.7, 28.6, and 6.7 %, respectively, whereas for sample B with nanoparticles, they are 9.0, 90.5, and 0.5 %, respectively. These results clearly indicate that sample A with nanoparticles is more highly cross-linked and contains fewer free polymers than those in sample B with nanoparticles. These tendencies are same as the data without nanoparticles shown in Fig. 7.

From these results, it can be concluded that the effects of the nanoparticles were limited and did not affect the discussion in this study.


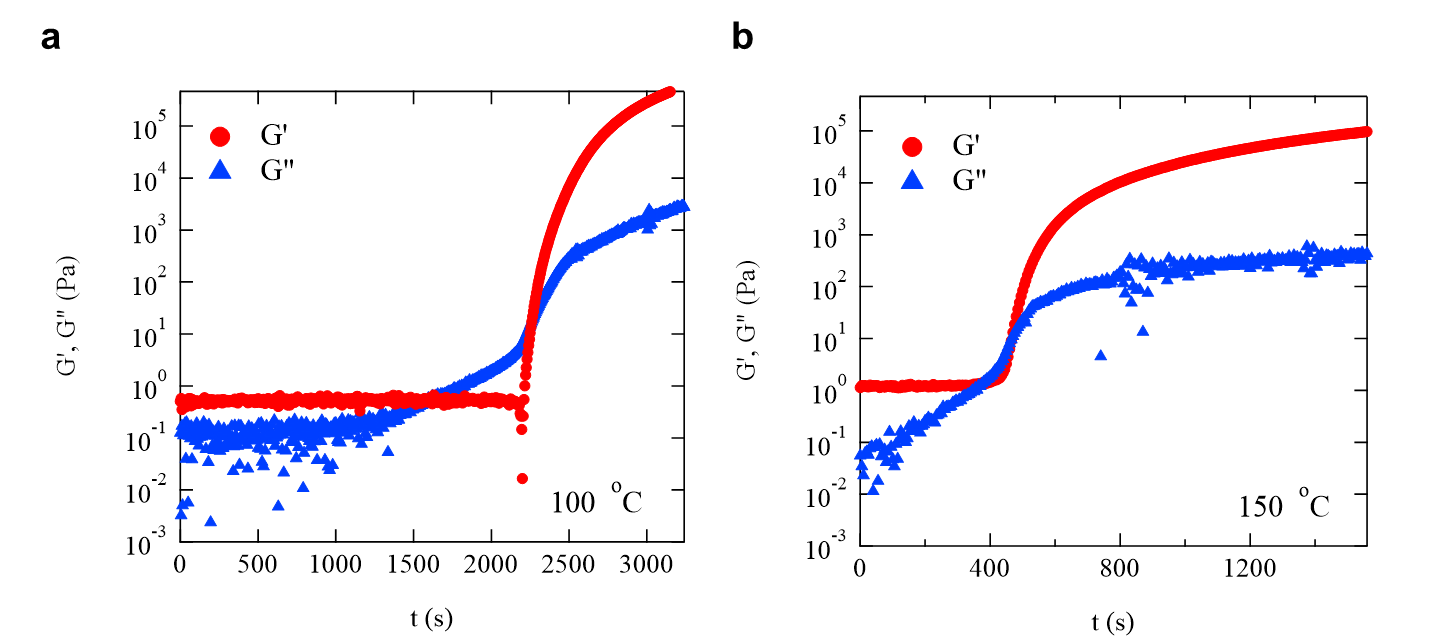


**Supplementary Fig. 9:** Time dependence of the storage elastic modulus $G'$ and the loss elastic modulus $G''$ obtained from rheometry during curing process of the epoxy resins with nanoparticles (1 vol%) at 100 °C (**a**) and 150 °C (**b**). The measurements were performed using a Malvern KINEXUS lab+ by the parallel plate rheometry method.

a

b

c

**Supplementary Fig. 10:** ^1^H-pulse nuclear magnetic resonance (NMR) spectrum for sample A with nanoparticles (the solid epoxy resin cured at 100 °C for 2 h and 150 °C for 5 h) (**a**). ^1^H-pulse NMR spectrum for sample B with nanoparticles (the solid epoxy resin cured at 150 °C for 5 h) (**b**). Both measurements were carried out at 120 °C. The solid lines in **a** and **b** are obtained from the fitting analysis with Eq. (5). **c** The molar fraction of protons for the hard polymer in the highly cross-linking part, the soft polymer in the poorly cross-linking part, and the free polymer obtained from the fitting analysis with Eq. (5).
